# Carotid Artery Dissection After Cervical Traction Device Use: A Case Report

**DOI:** 10.5811/cpcem.64694

**Published:** 2026-10-26

**Authors:** Kian D. Samadian, Maureen K. Nothnagle, Sina M. Mostaghimi, Sandra Alhoyek, Rehmat Babar, Shuhan He

**Affiliations:** * Massachusetts General Hospital, Department of Emergency Medicine, Boston, Massachusetts; † Brigham and Women’s Hospital, Department of Emergency Medicine, Boston, Massachusetts; ‡ Harvard Medical School, Boston, Massachusetts; § Massachusetts Eye and Ear, Department of Emergency Medicine, Boston, Massachusetts; ¶ Massachusetts Eye and Ear, Department of Ophthalmology, Boston, Massachusetts; || Massachusetts General Hospital, Department of Neurology, Boston, Massachusetts; ** Brigham and Women’s Hospital, Department of Neurology, Boston, Massachusetts; †† Present affiliation: University of Pennsylvania, Department of Emergency Medicine, Philadelphia, Pennsylvania

**Keywords:** internal carotid artery dissection, Horner syndrome, cervical traction device, carotid artery injury, case report

## Abstract

**Introduction:**

Internal carotid artery (ICA) dissection is a time-sensitive vascular emergency that may present with subtle findings, most notably painful Horner syndrome. Mechanical stress to the cervical vasculature is a recognized trigger for this condition.

**Case Report:**

We report a 56-year-old man who developed ipsilateral ptosis, miosis, and head and neck pain after using a home cervical traction device. Computed tomography angiography showed focal critical stenosis of the distal cervical left ICA consistent with dissection, along with a contralateral pseudoaneurysm suggesting prior injury. He was treated with dual antiplatelet therapy.

**Conclusion:**

This case highlights a potential association between cervical traction device use and carotid dissection and underscores the importance of recognizing painful Horner syndrome and bilateral carotid pathology, while prompting clinicians to specifically inquire about traction device use.

## INTRODUCTION

Carotid artery dissection is an important cause of stroke in adults younger than 60 years and frequently presents initially to the emergency department (ED).^1^–^6^ Internal carotid artery (ICA) dissection may manifest with cerebral ischemia, but a substantial subset of patients presents with isolated local symptoms, most commonly unilateral head or neck pain and partial Horner syndrome. Horner syndrome results from disruption of the oculosympathetic pathway. In the setting of ICA dissection, the lesion typically affects sympathetic nerve pathways traveling along the carotid artery, producing ipsilateral ptosis and miosis without facial anhidrosis. Painful Horner syndrome is classically associated with carotid dissection and should prompt urgent vascular imaging. Mechanical forces applied to the cervical spine, including manipulation and traction, can generate shear stress along the carotid artery.

Home cervical traction devices are widely marketed for spinal alignment and neck pain relief and apply sustained axial distraction forces. The vascular safety profile of these devices remains poorly characterized. This report aims to describe a case of ICA dissection presenting as painful Horner syndrome following use of a cervical traction device and to highlight key diagnostic considerations for emergency clinicians. This case report was prepared in accordance with the CARE (CAse REport) guidelines.^7^ Written informed consent was obtained from the patient for publication of this case report and accompanying images.

## CASE REPORT

A 56-year-old man with a history of benign prostatic hyperplasia, gastroesophageal reflux disease, asthma, allergic rhinitis, and cataracts was transferred from an ophthalmologic ED after computed tomography angiography (CTA) of the head and neck demonstrated a left ICA dissection during evaluation of a headache and drooping of the left eyelid. Approximately two weeks before presentation, the patient used an adjustable cervical neck traction device intended for spinal alignment and neck pain relief. Immediately after performing a cervical traction stretch, he developed a new left-sided headache. Over the ensuing week, he noted intermittent left posterior head and neck pain and progressive swelling and drooping of his left eyelid. He denied focal weakness, numbness, dysarthria, dysphagia, aphasia, or visual loss.

Three days before transfer, he was evaluated at an ophthalmologic ED, where ptosis was not documented. He returned three days later because of persistent left eyelid drooping. He denied high-velocity chiropractic manipulation or major trauma. On arrival to the ED, vital signs were notable for the following: blood pressure, 161/95 mm Hg; heart rate, 67 beats per minute; and peripheral oxygen saturation, 100% on room air. He was afebrile. On presentation, the patient was well-appearing and in no acute distress. Head, ears, nose, and throat examination was unremarkable, with an atraumatic head, a patent oropharynx, and normal tympanic membranes bilaterally. Ophthalmologic examination demonstrated anisocoria, with the left pupil smaller than the right, although both pupils were round and reactive to light. Administration of 0.5% apraclonidine ophthalmic drops resulted in reversal of the anisocoria with dilation of the left pupil and improvement in ptosis, confirming sympathetic denervation consistent with Horner syndrome. Cardiovascular examination revealed a regular rate and rhythm, and the lungs were clear to auscultation bilaterally. The abdomen was soft, nondistended, and nontender.

Neurologic examination demonstrated that the patient was alert and oriented with fluent speech and no evidence of dysarthria or aphasia. Cranial nerves II through XII were intact aside from the left-sided ptosis and miosis noted on ophthalmologic examination. Motor strength was full throughout, sensation was intact to light touch, finger-to-nose testing was normal bilaterally, and there was no pronator drift. Gait was normal.

*CPC-EM Capsule*

What do we already know about this clinical entity?

*Carotid artery dissection is a cause of painful Horner syndrome and ischemic stroke, often following trauma or cervical manipulation.*

What makes this presentation of disease reportable?

*This case links home cervical traction device use with carotid artery dissection, an uncommon and underrecognized mechanism of vascular injury.*

What is the major learning point?

*Painful Horner syndrome after cervical traction should prompt urgent vascular imaging to evaluate for carotid artery dissection.*

How might this improve emergency medicine practice?

*Recognizing cervical traction as a risk factor may expedite diagnosis, treatment, and stroke prevention in patients with subtle neurologic findings.*

Computed tomography angiography of the head and neck demonstrated short-segment luminal irregularity with focal critical stenosis in the distal cervical left ICA at the skull base, consistent with dissection, and a 0.9-cm pseudoaneurysm in the distal cervical right ICA, likely reflecting prior arterial injury (Images 1 and 2).

The patient reported surprise that a home neck traction device could be associated with a vascular injury and expressed appreciation for the timely diagnosis and management. He stated that he would avoid similar devices in the future. Neurology recommended initiation of dual antiplatelet therapy with aspirin and clopidogrel. At outpatient neurology follow-up, the patient remained neurologically intact without evidence of ischemic complications. His symptoms were stable, and he continued dual antiplatelet therapy. Further vascular imaging and longitudinal follow-up were planned.

## DISCUSSION

This case describes ICA dissection presenting as painful Horner syndrome following use of a home cervical traction device. The differential diagnosis of painful Horner syndrome includes pathology involving any portion of the three-neuron oculosympathetic pathway. Central etiologies include lateral medullary infarction, demyelinating disease, and brainstem tumors. Preganglionic lesions include apical lung tumors, mediastinal masses, cervical trauma, and postoperative injury. Postganglionic causes include ICA dissection, carotid artery aneurysm or pseudoaneurysm, cavernous sinus pathology, cluster headache, and orbital tumors. Additional considerations for unilateral ptosis with anisocoria include third cranial nerve palsy, pharmacologic pupillary abnormalities, and physiologic anisocoria. Ipsilateral neck pain and reversal of anisocoria with apraclonidine supported Horner syndrome. The temporal relationship between cervical traction, headache onset, and subsequent ptosis and miosis suggests a mechanically induced vascular injury.

Recognition of Horner syndrome in the ED carries significant diagnostic weight. In carotid dissection, disruption of postganglionic sympathetic fibers along the ICA produces ipsilateral ptosis and miosis without facial anhidrosis. Unilateral head or neck pain with new-onset ptosis or anisocoria should prompt evaluation for carotid dissection.^8^–^11^ Many patients lack focal deficits, and stroke may develop later due to thromboembolism. Early diagnosis allows timely antithrombotic therapy and reduces ischemic risk. Imaging revealed both an acute left ICA dissection and a contralateral 0.9-cm pseudoaneurysm. Bilateral carotid pathology is clinically significant, suggesting either sequential dissections from mechanical stress or underlying arterial vulnerability. The distal cervical ICA near the skull base is particularly susceptible to mechanical stress due to its mobility and tethering. Axial traction may increase shear forces along the arterial wall, leading to intimal injury, intramural hematoma, and pseudoaneurysm formation.^12^–^13^ The lesion location in this case corresponds to a region vulnerable during traction.

Our report complements two recent published reports describing cervical arterial injury after handheld massage gun use and bilateral carotid artery dissection following blunt trauma.^14^–^15^ Unlike these reports, our patient developed ICA dissection after use of a commercially available home cervical traction device without preceding high-energy trauma or chiropractic manipulation. As these devices become increasingly accessible to the public, emergency physicians should recognize home cervical traction as another potential mechanism of cervical vascular injury.

This report has limitations. As a single case, causation cannot be definitively established. However, the temporal sequence, focal dissection at a mechanically susceptible site, and absence of major trauma support a device-related mechanism. The contralateral pseudoaneurysm also raises concern for prior subclinical injury.

## CONCLUSION

This case reinforces a key emergency medicine principle: painful Horner syndrome should prompt immediate evaluation for carotid artery dissection. It also highlights a potential association between cervical traction devices and vascular injury. As these devices become more accessible, clinicians should maintain a high index of suspicion and obtain a targeted history to identify possible mechanical triggers.

## Figures and Tables

**Image 1:**
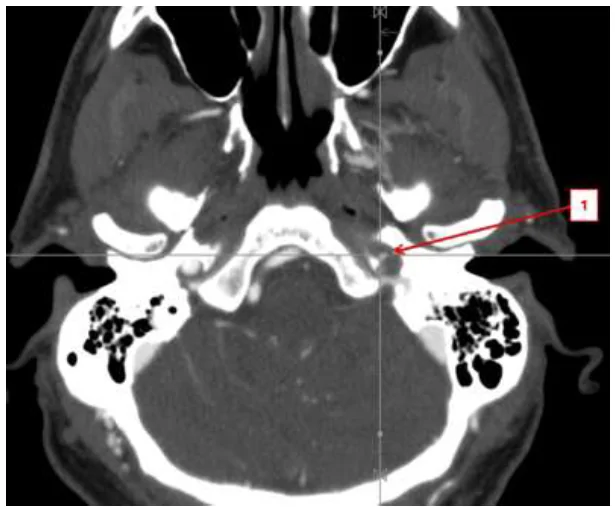
Computed tomography angiography of the head and neck demonstrating short-segment luminal irregularity with focal critical stenosis in the distal left internal carotid artery at the skull base (arrow), consistent with dissection.

**Image 2:**
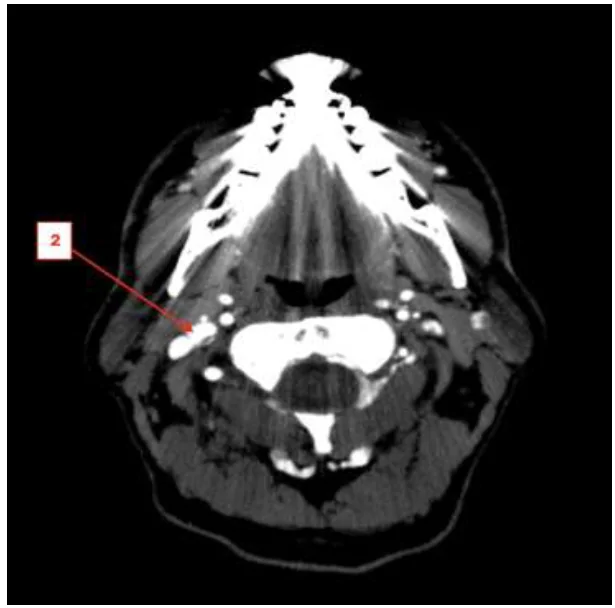
Computed tomography angiography of the head and neck demonstrating a 0.9 centimeter pseudoaneurysm in the distal cervical right internal carotid artery (arrow), likely reflecting prior arterial injury.
